# Development and validation of an interview guide for examining the effects of sports careers on the quality of life of retired Portuguese football players

**DOI:** 10.3389/fpsyg.2024.1374784

**Published:** 2024-03-12

**Authors:** Eduardo Teixeira, Carlos Silva, António Vicente

**Affiliations:** ^1^Sport Sciences School of Rio Maior, Polytechnic Institute of Santarém, Rio Maior, Portugal; ^2^Life Quality Research Centre, Polytechnic Institute of Santarem, Santarém, Portugal; ^3^Faculty of Human and Social Sciences, University of Beira Interior, Covilhã, Portugal

**Keywords:** football (soccer), interview guide, former players, post-career, career impacts, well - being

## Abstract

**Introduction:**

Considering the importance of assessing the impacts resulting from a sport career, this study aimed at developing and validating a semi-structure interview guide suitable for quantifying the sociodemographic and epidemiological profile of former professional football players.

**Methods:**

Based on the theoretical frameworks and several methodological procedures, an interview guide was developed, consisting of 3 areas of conceptual organization (A1. Biographical data; A2. Professional Career; and A3. Post-Career Transition) and 8 data collection categories (C1. Personal data; C2. Professional data; C3. Sociodemographic background; C4. Epidemiological pathway; C5. Moment of career retirement (career transition); C6. Post-career sociodemographic pathway; C7. Post-career epidemiological pathway; and C8. Perceptions of post-career planning). Thus, in procedural terms, four stages were considered for the construction and validation of the interview guide, namely the Ad hoc construction of the interview guide (i), the review of the in-terview guide by a panel of experts (ii), definition of procedures and protocol for the application of the interview (iii), and the application of the pilot study for the face validation of the interview guide (iv). The sample consisted of two former Portuguese professional players.

**Results and discussion:**

To analyze and discuss the data, a content analysis was carried out on all the answers given to each question in the script by the interviewees. From them, matrices were created with the response references to each subcategory. In this way, it was possible to analyse the type of answers given by the interviewees and relate them to the theoretical perspectives of the topic being investigated. The results showed that the interview guide for the study of the impacts of the sports careers on the quality of life of Portuguese former football players shows reliability for the collection of qualitative and quantitative information from the respective content analysis. The use of the interview guide characterizes the path of former player, providing information and knowledge on the sociodemographic and epidemiological impact factors resulting from their post-career.

## 1 Introduction

In recent decades, there has been a significant increase in research focused on understanding the long-term impacts of sports careers (Stambulova and Wylleman, 2019; Stambulova et al., 2021; Samuel et al., 2023).

Career transition for athletes is a pivotal juncture in their lives, occurring at an early stage compared to individuals in the general population, non-athletes (Vilanova and Puig, 2017; Wylleman, 2019; Yao et al., 2020). This timing complexity and adds layers of complexity to the process, making it a multidimensional, multilevel, and multifactorial experience (Martin et al., 2014). Consequently, there is an urgent imperative to cultivate specialized and interdisciplinary knowledge from various scientific domains to gain insights into a phenomenon that has far-reaching impacts on both society as a whole and individuals. In the realm of football, sports careers are marked by significant physical strain, considering the extensive depletion of motor energy resources (Barreira et al., 2015; Carapinheira et al., 2019; Silva et al., 2022; Nunes et al., 2023).

Engaging in the sport at the professional level comes with a heightened risk of injury (Roos, 1998; Ekstrand et al., 2011; Pfirrmann et al., 2016; Zech and Wellmann, 2017). Concurrently, the time constraints of an athlete’s career compel them to form a deeper connection with their profession, encompassing matters like daily availability, the pursuit of enhanced earnings, the sacrifices of social life, dietary habits, and other facets that may not be as conspicuous in non-sporting occupations (Smismans, 2022). In addition to these considerations, football players often assume a role of social prominence, leading to their recognition and characterization through various descriptors. Moreover, this recognition situates them within a limited social circle (Drawer and Fuller, 2002; Arliani et al., 2016; Monteiro et al., 2021).

A career can be defined as a sequential series of attitudes and behaviors tied to an individual’s experiences and activities related to their work over the course of their life (Hall, 2002). It represents a continually evolving process, consisting of distinct stages, each with its specific demands (Schlossberg, 1981; Wylleman et al., 1993; Wylleman, 2019). One of these stages is the transition to post-career life. In the context of football, this transition signifies the moment when a player departs from their professional career and enters a new phase of life (Wylleman et al., 1993; Stambulova et al., 2009, 2021). This situation is particularly delicate as it necessitates the player’s emotional adaptation to a new social environment, a different status, and an altered lifestyle (Brandão et al., 2000; Kuettel et al., 2017).

The number of studies in this field, especially in football, is relatively limited. Therefore, it appears pertinent to identify, collect, and disseminate data related to various aspects such as the activity process, professional retirement, sociodemographic realities, health consequences, and longevity influenced by the careers of footballers, guided by existing literature (Rintaugu, 2011; Knights et al., 2016, 2019; Carapinheira et al., 2018a, 2019). To gain a more precise understanding of the patterns and requirements of these professionals, it is essential for research to concentrate on the specific sport and, if feasible, facilitate cross-cultural comparisons (Stambulova and Samuel, 2019; Stambulova et al., 2021). Building on theoretical models concerning careers and career transitions (Taylor and Ogilvie, 1994; Stambulova, 2003; Wylleman et al., 2004; Stambulova et al., 2006; Creswell and Poth, 2017;Wylleman, 2019; Stambulova et al., 2021), a research trajectory has emerged with the objective of examining the sociodemographic and epidemiological effects stemming from the professional football career of former players. Taking this viewpoint into consideration and employing qualitative methodology, we have developed and confirmed the validity of a semi-structured interview guide. This approach, in accordance with the perspectives of multiple authors (Wolcott, 1994; Gall et al., 2007; Turner, 2010; Creswell and Poth, 2017), is an established research method for acquiring comprehensive insights into participants’ experiences, emotions, and interpretations.

Framed within the research paradigm on the theme of careers and sport transitions, specifically oriented toward research on the impacts resulting from the professional football career and its effects on the perception of post-career quality of live, as well as in the pursuit of scientific studies carried out in this area by several authors (Drawer and Fuller, 2001; Agresta et al., 2008; Curran, 2015; Sanders and Stevinson, 2017; van Ramele et al., 2017; Carapinheira et al., 2018b; Nunes et al., 2023), the main purpose was to build and validate a meta-evaluative instrument, in the form of a checklist, focused on the interpretivist paradigm, capable of characterizing the sociodemographic and epidemiological profile of former football players in Portugal, considering all the transitional phases of the career, namely from the beginning of the professional activity to the situational moment in the post-career.

The interview guide aims to description of career development stages and career pathways with prediction of normative transitions (Samela, 1994; Wylleman, 2019; Samuel et al., 2023). Simultaneously, aimed at description and explanation of a transition process and factors involved in terms of normative, non-normative and quasi-normative athletic transitions (Taylor and Ogilvie, 1994; Stambulova, 2003; Ryba et al., 2016).

The purpose of applying this interview is to develop several studies that holistically conceptualize the entire journey of former football players as a way of evaluating the career impact factors and the quality of life of this population in the post-career period. Used to this end, collecting data from this instrument will provide information on the sociodemographic and epidemiological idiosyncrasies experienced during the sport career, career transition and post-career.

The main objective of this study was to validate the Interview Guide for Examining the Effects of Sports Careers on the Quality of Life of Retired Portuguese Football Players. From the pilot study, we seek to ensure that the interview is carried out in an organized, methodical manner and, simultaneously, that the information collected is framed and directed to each area and specific thematic category. To this end, matrices will be created to characterizing all types of data to be collected through the application of the instrument.

## 2 Materials and methods

Before initiating the construction of this interview guide, the ethical committee at the University of Beira Interior was obtained (CE-UBI-Pj-2021-015). The quality assurance of an interview for application in the scientific field should respect a set of methodological procedures (Wolcott, 1994; Flick, 2005; Boni and Quaresma, 2005). The theoretical framework, the choice of the type of interview, the interview structure definition, the relevance and clarity of the questions, the validation process, the interview training, the interview application and the data processing are some of the steps highlighted in the literature (Triviños, 1987; Wolcott, 1994; Flick, 2005; Boni and Quaresma, 2005; Aires, 2015).

Drawing upon the theoretical framework, which is informed by a comprehensive review of the literature concerning the effects of former footballers’ professional careers, particularly within sociodemographic and epidemiological dimensions, we meticulously developed a semi-structured interview guide tailored to the research objectives. We opted for a semi-structured interview approach due to its capacity to incorporate relevant theories and hypotheses related to the research theme. This approach not only facilitates the description of social phenomena but also enables their comprehensive explanation and understanding (Triviños, 1987; Aires, 2015).

In terms of the procedural aspects, four distinct stages were considered for the creation and validation of the interview guide: (i) the *ad hoc* construction of the interview guide, (ii) a critical review of the interview guide by a panel of experts, (iii) the formulation of procedures and a protocol for administering the interview, and (iv) the execution of a pilot study to assess the face validity of the interview guide.

### 2.1 *Ad hoc* construction of the interview guide

To begin, we initiated our research process by exploring scientific articles centered about careers and post-career transitions in sports, with a particular emphasis on football. We conducted these searches using relevant keywords within popular search engines. Subsequently, we scrutinized the methodologies employed in both quantitative and qualitative studies that delved into career perspectives and career transitions within a sociodemographic and epidemiological framework. The intention was to utilize this analysis to structure the interview guide into thematic sections, aligning them with the overarching research objectives. This process was informed by insights from various sources (Agresta et al., 2008; Knights et al., 2016; Zech and Wellmann, 2017; Carapinheira et al., 2018b; Lelbach et al., 2020).

It is widely acknowledged that conceptual research on career transitions should embrace a comprehensive, continuous, and multifaceted approach (Taylor and Ogilvie, 1994; Wylleman et al., 2004; Stambulova et al., 2009). This entails recognizing the unique challenges that athletes face at different stages of their sporting journey, as well as in other aspects of their lives, including their interactions with coaches, parents, and friends (Côté, 1999; Wylleman et al., 2000; Côté et al., 2007). Moreover, it necessitates an understanding of the influence of macrosocial factors such as culture, context, and the personal development of the player (Stambulova et al., 2007). With this perspective in mind, the initial version of the interview guide was structured around three distinct areas, drawing support from relevant theoretical models (Taylor and Ogilvie, 1994; Stambulova et al., 2006; Wylleman, 2019). Subsequently, after considering insights from various sources (Turner et al., 2000; Drawer and Fuller, 2001; Uzunca et al., 2005; Agresta et al., 2008; Baron et al., 2012; Vann Jones et al., 2014; Gomes and Domingues, 2016; Gledhill et al., 2017; van Ramele et al., 2017; Carapinheira et al., 2019; Yao et al., 2020; Jones et al., 2021), we made the decision to incorporate eight research categories, each containing numerous questions organized into various subcategories.

As an initial premise, meticulous attention was given to the wording, structure, and sequence of the questions to ensure that they were framed objectively, in plain language, and without inadvertently suggesting any specific answers (Wolcott, 1994).

### 2.2 Evaluation of the interview guide by an expert panel

Following the creation of the initial draft of the interview guide, it underwent a face validation process by a panel of three experts from different institutions of Portuguese university institutions. These experts were experienced higher education teachers with a track record of research and published work in the relevant field (Scorsolini-Comin, 2020). After receiving their feedback, several modifications were made to the interview guide. Specifically, eight questions were removed, and the wording of seven other questions was revised, while keeping intact the originally defined categories and subcategories.

### 2.3 Procedures and protocol for applying the interview

To ensure a thorough and meticulous utilization of the instrument, a set of procedures was established for both preparation and implementation, particularly with regard to the form of contact and preparation for the interview (i. selection and provision of contacts; ii. checking participant availability; iii. Information about the scope and objectives of the research; iv. schedule for the day and location of the interview; v. preparation of a model with the interviewee’s pre-defined CV) and, also, the development of a protocol covering the pre-interview, during-interview, and post-interview phases (i. explanation of the scope, objectives and organization of the interview; ii. clarification on the dissemination of results and their confidentiality; iii. Authorization to record the interview; iv. display of the summary of the interviewee’s CV to confirm or change the information; v. reading of informed consent for subsequent signing in duplicate of the document; vi. definition of the starting and ending moments of the interview; vii. Reinforcement of the possibility of providing relevant information not mentioned during the interview; viii. Information about the possibility of providing the interview transcript; ix. final acknowledgment).

### 2.4 Execution of the pilot study

To assess the practical effectiveness of the interview we conducted a pilot study, including factors like its flow, duration, question clarity, and any unforeseen issues (Gall et al., 2007). The primary objective was to identify any constraints or shortcomings in the interview structure (Turner, 2010). The pilot study involved a sample of two retired Portuguese football players, each having accumulated over 8 years of professional experience.

Due to the constraints imposed by the SARS-CoV-2 pandemic, the interviews were conducted by videoconference (platform Zoom). This approach was chosen to maintain a desirable level of comfort for the interviewees (Wolcott, 1994; Boni and Quaresma, 2005). It allowed them to participate from the comfort of their own environment while interacting with the interviewer in a quiet, confidential setting and having easy access to the interview guide. On average, the interviews lasted approximately 42 min, which was considered an appropriate duration for field application. Additionally, this phase served as a training opportunity for the interviewer, helping to assess the relevance of the topics covered and evaluate the clarity of the questions to eliminate any potential comprehension issues (Gomes and Domingues, 2016). In conclusion, the final version of the interview guide comprises three main areas and eight categories, encompassing a total of 27 questions more a general questionnaire with biographical and professional data.

#### 2.4.1 Interviews transcription

The interviews were recorded in audio and video format and transcribed. The QSR International – Nvivo, version 11.0 software and SPSS Statistics was used for the information analysis and processing (Leitão, 2002; Bardin, 2007). Simultaneously, to make inferences and interpretations regarding the content analysis, the answers given by each interviewee were detailed, through a table, for each of the categories and subcategories identified in the interview guide.

#### 2.4.2 Content analysis of the interviews

The social construction of an instrument must always be guided by the theoretical framework of the research (Bardin, 2016). Concomitantly, content analysis is a well-established qualitative research method used to interpret the meaning within textual content, aligning with the naturalistic paradigm (Scorsolini-Comin, 2020).

In this sense, the content analysis respected the different phases of analysis, namely pre-analysis (organization), exploration of the material (coding, categorization) and treatment of results (inference and interpretation) (Bardin, 2016). The validation of the interviews was conducted through conventional content analysis, wherein coding areas and categories were directly derived from the textual data. This approach aimed to extract meaning from the transcribed content by organizing it into discrete sections of information, each identified by specific codes (Creswell and Poth, 2017). By employing this method, we ensured the validation of the discourse by the individuals providing the information and upheld the quality of the procedural dialog inherent in the interview technique (Turner, 2010).

To define the codes for presenting and discussing the results, we analyzed phrases, expressions, and recurring ideas common among the selected interviewees. This process helped identify thematic categories and subcategories based on the interview protocol and the primary topics addressed during the interviews. Subsequently, a review of the initial structure of these categories and subcategories was undertaken, incorporating new criteria as additional response options emerged. This approach aligns with recommendations from other authors (Bardin, 2007) for a mixed methodology that simultaneously utilizes pre-defined categories and adapts to new subcategories that best suit the analyzed material (Creswell and Poth, 2017; Creswell and Plano, 2017).

At the same time, for which answer former players gave, argumentative and guidance lists were created about the way which question should be led, so that the quality and pertinence of the data in which category could be satisfied.

## 3 Results and discussion

The methodology employed, stemming from the content analysis conducted during the research, involved constructing a comprehensive matrix of responses for each of the categories outlined in the guide. This matrix facilitated the identification of all the subcategories and criteria that emerged. Subsequently, these findings were discussed in the context of the theoretical framework pertaining to careers and sports transitions.

### 3.1 Biographical data (area 1)

**Figure 1:**
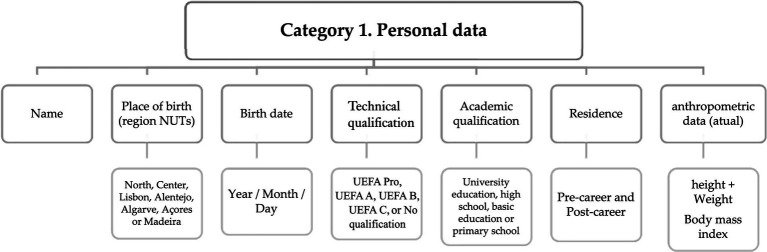
Matrix with codes for personal information.

The collection of informative biographical data is common practice among severel retrospective studies (Drawer and Fuller, 2001; Vann Jones et al., 2014; Arliani et al., 2016; Carapinheira et al., 2019; Stambulova and Wylleman, 2019; Jones et al., 2021; García-García et al., 2023; Sun and Moon, 2023). In this regard, and firstly, through closed questions, the personal data of the former players were collected (Figure 1).

**Figure 2:**
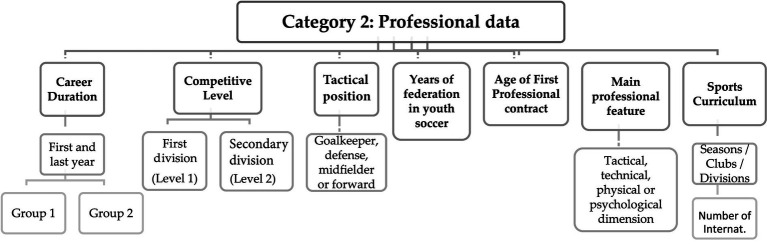
Matrix with response codes related to the career trajectory.

Both closed and open-ended questions were employed to enable the subsequent determination of dependent and independent variables based on the objectives and research questions posed in scientific studies. This approach aimed to enhance the identification and characterization of former players in relation to their professional careers (Figure 2).

The analyzed content revealed that the responses led to a distinct identification of the criteria for each of the subcategories. The interviewees displayed clarity in their understanding of the questions posed to them, facilitating the interpretation of each subcategory during the transcription of the interviews.

The essentially quantitative information collected in these two categories allow for the personal and professional characteristics of the interviewees.

### 3.2 Professional career (area 2)

The domain of professional careers encompasses two categories, each providing retrospective information about the sociodemographic and epidemiological aspects of former football players’ careers. These categories aim to clarify the unique attributes of football players’ careers, particularly within a systems perspective, which will be analyzed in the context of sports psychology (Stambulova et al., 2021). From the career models proposed by Stambulova (Stambulova, 2003) we opted for the guideline of the structural model, i.e., to develop questions that could originate data concerning the direction of sport development (e.g., motivation, quality and style) and, simultaneously, of the psychological determinants reflecting aspects of operational (e.g., psychological processes triggered), situational (e.g., activities and behaviors) and cultural (e.g., organization and lifestyle) scope.

**Figure 3:**
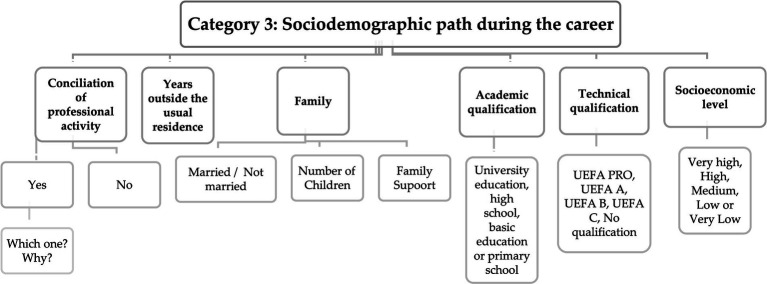
Matrix with response codes regarding the sociodemographic path during the career.

Through the content analysis of interviews pertaining to category 3, which is focused on the sociodemographic aspect, six distinct subcategories have emerged as crucial for exploring and gathering information. These subcategories include the ability to balance professional commitments, the duration of time spent away from one’s native area of residence, changes in family dynamics, the pursuit of academic or technical education, and the perceived socio-economic status achieved during the career. These category are visually represented in Figure 3.

**Figure 4:**
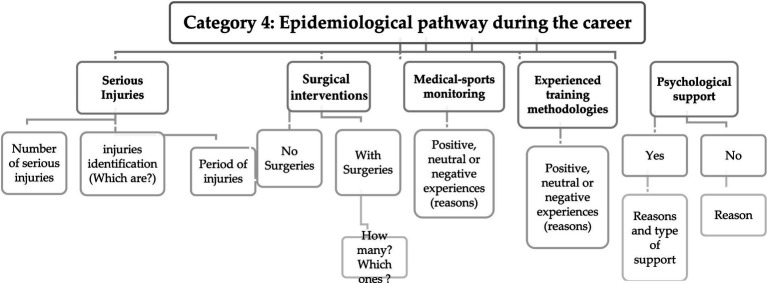
Matrix with response codes regarding the epidemiological pathway during the career.

Regarding the content analysis of interviews in category 4 (see Figure 4), we have identified and grouped five subcategories for extracting relevant information. These subcriteria were designed to collect data concerning the occurrence of significant injuries, any surgeries undergone, assessments of the quality of sports medical care, opinions on methodological experiences, and the availability of psychological support during the players’ careers. These criteria are aimed at investigating whether health issues experienced during the professional career are indeed factors that should be considered in assessing the quality of life for individuals’ post-career. This aligns with findings from previous research studies (Agresta et al., 2008; van Ramele et al., 2017; Lelbach et al., 2020).

Content analysis shows that there is agreement between the researchers in identifying the number and type of injuries that former players considered to be serious during their career. A similar pattern emerges concerning both the quantity and nature of surgeries undertaken, alongside the recognition of the presence of psychological support. The findings suggest that it could be valuable to categorize timeframes during which injuries and surgeries occurred. Regarding the perceptions of sports medical care and the methodologies encountered, coding presented challenges because former players indicated having diverse practices and experiences throughout their careers. This diversity arose from interactions with different medical departments and technical teams, which naturally influenced their perceptions of the quality of experiences. To address this issue and ensure more straightforward coding, both questions should initially be structured with closed-answer options. In other words, former players should assess these criteria as positive, neutral, or negative when considering their entire career trajectory. Subsequently, in a follow-up open-ended question, they can provide justifications and explanations for their choices.

### 3.3 Transition to post-career (area 3)

The category addressing the termination of a sports career drew inspiration from two key models: the conceptual model of adaptation to retirement among athletes (Taylor and Ogilvie, 1994; Kuettel et al., 2017) and the Sport Career Transition model (Stambulova, 2003). The questions posed inquired, from a comprehensive standpoint, about the variables that either influence or are associated with the professional career retirement process.

**Figure 5:**
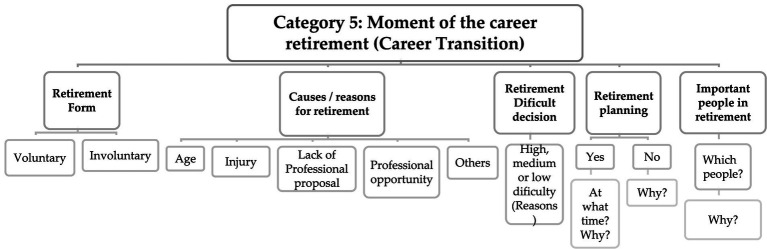
Matrix with response codes regarding the professional career retirement.

Through the results obtained (Figure 5), it seems that the contents of the answers present informative assumptions capable of inferring about the quality of the transition experienced by former players and, concomitantly, to understand which resources were used for this transition, which results in the effective identification of dependent variables t that define the conclusion of the careers within this population (Park et al., 2013; Carapinheira et al., 2018b; Samuel et al., 2023). The factors that determinate the quality of the transition to post-career are the voluntariness (or involuntariness) of the retirement, the time of acceptance, the athletic identity, the new professional orientation, and the life changes that arise at the moment of retirement. On the other perspective, and in relation to the resources used for this transition, the existence of coping strategies, the type of psychological support, the existence (or not) of a career retirement plan and also the use (or not) of support from programs are highlighted (Carapinheira et al., 2018b).

From the content analysis it is possible to identify the most significant positive and negative factors of transition quality and the resources available for the players which, by itself, helps in the implementation of specialized intervention models by levels (i.e., cognitive, behavioral, emotional, and social) that have purpose of helping athletes who had negative transitions (Wylleman et al., 2000; Sofie et al., 2021; Smismans, 2022). A higher degree of athletic identity related to valued sport correlates such as intrinsic motivation and the mastery goal orientation (Lochbaum et al., 2022) which means that the type of answers given by the interviewees can be analyzed in light of this paradigm.

**Figure 6:**
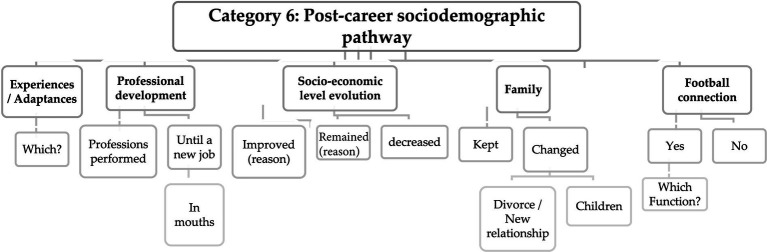
Matrix with response codes regarding post-career sociodemographic pathway.

**Figure 7:**
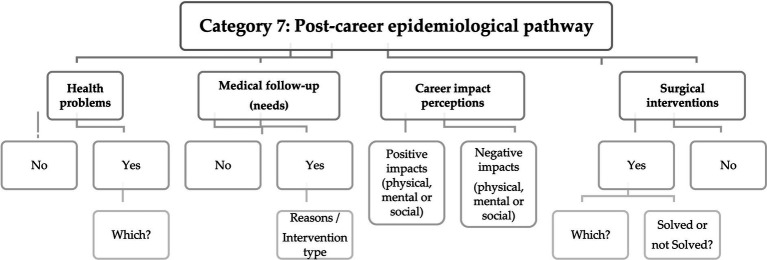
Matrix with response codes for the post-career epidemiological pathway.

However, building upon the concept that career and sport transitions should be examined considering the athlete as a holistic entity within a specific context (Stambulova et al., 2021), the answers given in category 6 and 7 (Figures 6,7) emphasize the quality factors and resources that are integral to the transition process, as well as they define the subsequent path of former football players post-career.

For the characterization of sociodemographic variables, the answers showed descriptions related to the experiences and adaptations in post-career period, the professional path, the changes at socio-economic level, the dynamics and development of the family, and the continuity (or discontinuity) related to the connection that former players maintained with football, as described and proposed in other studies (Grove and Stoll, 1999; Uzunca et al., 2005; Agresta et al., 2008; Rintaugu, 2011; Gazzaroli et al., 2017; Carapinheira et al., 2018b; Monteiro et al., 2021).

It’s possible that the content of these questions could provide relevant information’s because adaptive processes occur within specific contexts that may influence former sport performers’ responses and outcomes (Schinke and Stambulova, 2017).

In the epidemiological characterization, the results allow us to recognize if there are (or have been) health problems, medical follow-up and medical-surgical interventions in the post-career period, aspects that are also highlighted in several scientific papers reviewed (Drawer and Fuller, 2001; Uzunca et al., 2005; Park et al., 2013; Vann Jones et al., 2014; Arliani et al., 2016; Sanders and Stevinson, 2017; Jones et al., 2021).

At the same time, by analyzing the criteria concerning perceptions of career impact, we gather the former players’ overarching views regarding the positive and/or negative effects linked to their professional careers, encompassing physical, mental, and social dimensions. So, It will be important aimed to summarize the post-career effects of highly competitive football practice.

It was possible to develop the matrices proposed in Figure 7, where the possible response codes are presented. Through content analysis, the type of answers given by the interviewees allow us to understand the health problems that occurred in post-career, whether former players needed support or medical follow-up, whether there were surgical interventions and, especially, in the case occurrence of health problems, their perception of whether this could be related to their professional career.

In summary of the two categories (post-career sociodemographic and epidemiological pathway), the length of the adaptation process is a factor that can be investigating. Adaption is a dynamic process, the interplay between appraisals, decision-making and active coping may change depending on the course of the transition (Samuel et al., 2023).

In this regard, analyzing the sociodemographic and epidemiological information of former players during their retirement could provide clues about the impact of their careers, the planification of career transition and, at the same time, understand the profiles, life choices and the type of social, professional and health reality that these former players face.

The last category of the interview guide aims to gather the former players opinions regarding the importance of post-career planning, the knowledge and importance of support programs, their suggestions on the topic and, also, from a retrospective, the identification of key moments and possible addictions or dependencies experienced during their life path (Stambulova et al., 2009; Dimoula et al., 2013; Park et al., 2013; Carapinheira et al., 2018b; Jones et al., 2021; Samuel et al., 2023).

**Figure 8:**
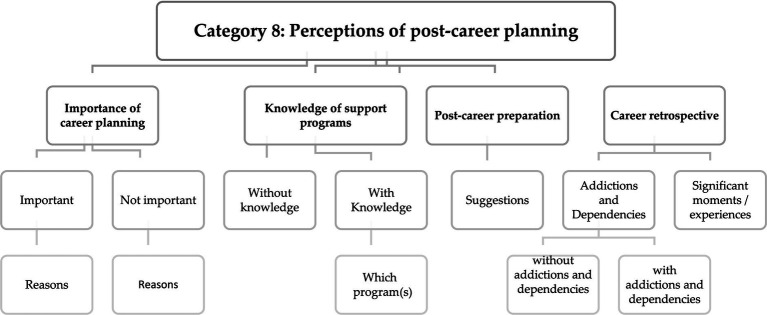
Matrix with response codes regarding former players perceptions of post-career planning.

From the content analysis of the research, it is possible to create response codes from four subcategories (Figure 8). In this perspective, it seems relevant to assess the set of opinions and suggestions of former professional players that may contribute to the transfer of recommendations to support programs for career transition (McKnight and Kashdan, 2009), in order to help players to get involved in life after sport, helping them to understand the skills they need to succeeded in other areas of activity. Adaptation and professional support associated with change events must consider age and identity as part of the process (Samuel et al., 2023).

The lives of sport performers have tremendously changed in last years as a result of the globalization process, social media, and migration (Samuel, 2021). Analyzing the responses from former players, we expect that the collection of information from these subcategories will bring new perspectives and visions about the strategies that can be adopted in this research area.

## 4 Conclusion

The use of the investigative technique of interview survey is an important research instrument that allows achieving a deep knowledge of the object of study, to the extent that it allows, based on the information provided by the interviewees, to (re) construct meanings and senses.

Under this premise, the objectives of this work aimed at the construction, development and validation of an interview guide based on several methodological procedures that would ensure a robust theoretical framework, a justification for the definition of the primary research areas and, through content analysis of two interviews with former football players, systematize the information model to be generated through the application of the interview (Table 1). Therefore, attached to this article is the complete interview guide with all the questions written in Portuguese language.

**Table 1:** Areas, categories, and subcategories of the interview guide.

Area 1: biographical data	
Category 1: personal data	Category 2: professional data
Full name / sport name Place of birth / date of birth Academic and thecnical qualifications Residency (pree-carreer and post-carreer) Height and weight (current)	Year of beginning and end of career Internationalisations / tactical position Start of federated practice (formation) Characteristics of professionalisation Curriculum vitae summary

Area 2: professional career	
Category 3: sociodemographic backgroud	Category 4: epidemiological pathway
Conciliation of professional activity Years of living outside the usual residence Family Academic and thechnical habilitations General socio-economic level	Serious injuries Surgical interventions Medical-sports monitoring Experienced training methodologies Psychological suport

Area 3: transition for post-career	
Category 5: moment of career retirement (carreer transition)	Category 6: post-career sociodemographic pathway
Retirement form Causes or reasons for retirement Retirement decision Retirement planning Important people in the retirement	Experiences and adaptations Professional development Socio-economic level evolution Family Football connection

Category 7: post-career epidemiological pathway	Category 8: perceptions of post-career planning
Health problems Medical follow-up (needs) Career impact perceptions Surgical interventions	Importance of post-career planning The knowledge of support programmes Post-career preparation (sugestions) Career retrospective (significant or problematic experiences)

The interview guide designed to investigate the impacts of a sports career on the quality of life of Portuguese former football players has proven effective in collecting both qualitative and quantitative data through content analysis (Table 2).

**Table 2:** Characterization of the interviewed individuals for the pilot study.

	Former player 1	Former Player 2
Career time	1983–1999	1982–1993
Tactical position	Midfielder	Defense
Competitive level	Level 1 (first division)	Level 2 (second division)

The instrument demonstrate efficiency as it delves deeply into the situational context of former players, facilitating a comprehensive understanding and interpretation of their experiences as conveyed by the diverse research participants. This is relieved in the matrices developed for each of the guide’s areas, categories, and subcategories. The instrument seems pertinent regarding data collection for the scope of research oriented toward the study of sociodemographic and epidemiological impacts arising from a professional football career, with transcultural characteristics, focused on individual and multidimensional perception in assessing quality of life in specific context.

## Data availability statement

The original data contributions presented are included in the Supplementary material. Further inquiries can be directed to the corresponding author.

## Ethics statement

The studies involving humans were approved by the Ethical committee at the University of Beira Interior (CE-UBI-Pj-2021-015). The studies were conducted in accordance with the local legislation and institutional requirements. The participants provided their written informed consent to participate in this study.

## Author contributions

ET: Conceptualization, Data curation, Formal analysis, Investigation, Methodology, Project administration, Resources, Software, Validation, Visualization, Writing – original draft, Writing – review & editing. CS: Data curation, Formal analysis, Software, Supervision, Validation, Visualization, Writing – review & editing. AV: Conceptualization, Investigation, Methodology, Supervision, Validation, Visualization, Writing – review & editing.
