# Supplementary material for: Development and validation of an interview guide for examining the effects of sports careers on the quality of life of retired Portuguese football players
Source: Front Psychol. 2024 Mar 12;15:1374784. doi: 10.3389/fpsyg.2024.1374784 (PMC10963604; doi:10.3389/fpsyg.2024.1374784)
Supplement: Supplementary file 1 [file Data_Sheet_1.PDF]

ENTREVISTA PARA O ESTUDO DOS IMPACTOS DA CARREIRA DESPORTIVA NA QUALIDADE DE VIDA DOS EX-JOGADORES DE FUTEBOL PORTUGUESES

GUIÃO

Estimado participante,

Eu, Eduardo Filipe Magalhães Teixeira, professor adjunto da Escola Superior de Desporto de Rio Maior, do Instituto Politécnico de Santarém, estudante de Doutoramento em Ciências do Desporto na Universidade da Beira Interior, orientado academicamente pelos Professores Doutores António Vicente (Universidade da Beira Interior) e Carlos Silva (ESDRM-IPS), e com a colaboração do estudante de Mestrado em Desporto João Santos, encontro-me a realizar um trabalho de investigação na área do futebol, nomeadamente um estudo sobre os impactos sociodemográficos e epidemiológicos decorrentes da carreira desportiva dos ex-jogadores profissionais de futebol portugueses.

Consideramos que o seu testemunho enquanto ex-jogador profissional de futebol pode contribuir para aumentar o conhecimento do real impacto sociodemográfico e epidemiológico da carreira. Concomitantemente, pensamos que a partir dos resultados obtidos nos vários estudos será possível contribuir para a construção/desenvolvimento de modelos que apoiem/auxiliem os jogadores e ex-jogadores profissionais a melhor prepararem a sua transição pós-carreira.

Neste sentido, vimos por este meio solicitar a sua colaboração numa entrevista que será realizada de acordo com um guião elaborado e validado para o efeito, juntamente com a aplicação de 1 questionário, nomeadamente o WHOQOL-BREF - World Health Organization Quality of Life – Bref (Canavarro et. all, 2006).

A recolha de dados através desta entrevista será registada de forma escrita e gravada (em formato áudio) pelo investigador principal (Eduardo Teixeira) ou por um entrevistador assistente (João Santos) que colabora diretamente com o investigador neste âmbito. Os dados recolhidos serão totalmente confidenciais, assegurando-se o seu anonimato, e serão utilizados apenas para os fins propostos na presente investigação.

Esta investigação cumpre com as normas em vigor previstas para a proteção de dados. As respostas são totalmente anónimas. Todas as respostas fornecidas serão armazenadas num banco de dados associado a um código alfanumérico. Os dados armazenados serão salvos por meio de uma senha à qual somente o investigador responsável terá acesso ficando este encarregue pela sua guarda e destruição no final do estudo imediatamente após a defesa do doutoramento. Nenhum dos membros da equipa ou participantes retirará deste estudo qualquer ganho económico. A sua participação é voluntária, não remunerada e pode desistir a qualquer momento, sem qualquer tipo de consequência.

O investigador assume a responsabilidade pelo desenvolvimento do estudo comprometendo-se a responder a qualquer dúvida. Para qualquer questão relacionada com a participação no estudo, por favor, contacte o seguinte e-mail: eduardoteixeira@esdrm.ipsantarem.pt (investigador principal).

Ao participar neste estudo, você confirma que:

- Recebeu informações suficientes sobre o estudo e foi-lhe dada a oportunidade de fazer perguntas sobre o mesmo;

- Entende que a sua participação é voluntária;

- Entende que os dados serão tratados de forma anónima e confidencial (sujeitos às garantias previstas na Lei n.º 58/2019 de 8 de agosto - Regulamento Geral de Proteção de Dados) e que serão usados apenas para fins da presente investigação e estudo sobre os impactos sociodemográficos e epidemiológicos decorrentes da carreira desportiva dos ex-jogadores profissionais de futebol portugueses;

- Entende que pode retirar-se/desistir do estudo em qualquer altura e sem qualquer consequência ou penalização.

Caso concorde com as informações prestadas, e se disponibilize para colaborar nesta investigação, agradecemos que assine este documento:

O ENTREVISTADO	O INVESTIGADOR	O ENTREVISTADOR

DATA ENTREVISTA	LOCAL ENTREVISTA

ÁREA 1: DADOS BIOGRÁFICOS

CATEGORIA I E CATEGORIA II – DADOS PESSOAIS E PROFISSIONAIS

**NOME COMPLETO:**	
**NOME DESPORTIVO (ou alcunha):**	
**DATA NASCIMENTO:**	
**NATURALIDADE:**	
**LOCAL DE RESIDÊNCIA (atual)**	
**LOCAL DE RESIDÊNCIA (pré-carreira):**	
**HABILITAÇÃO ACADÉMICA:**	
**HABILITAÇÕES TÉCNICAS:**	
**INÍCIO DA FORMAÇÃO (federado):**	
**INÍCIO CARREIRA PROFISSIONAL:**	
**Anos enquanto sénior para se tornar profissional**	
**TÉRMINO DA CARREIRA PROFISSIONAL:**	
**Nº DE INTERNACIONALIZAÇÕES:**	
**POSIÇÃO TÁTICA ONDE JOGAVA:**	
**CARACTERÍSTICA PRINCIPAL PARA A PROFISSIONALIZAÇÃO:**	
**DADOS ANTROPOMÉTRICOS (peso e altura):**	
Currículo Profissional*:	

Pré-preparação de currículo profissional (por parte do investigador) dos jogadores com identificação cronológica dos clubes em que jogou e dos respetivos níveis competitivos (campeonato/divisão) para ser confirmado e/ou ajustado no momento da aplicação da entrevista (incluindo o seu percurso no futebol de formação).

ÁREA 2: A CARREIRA PROFISSIONAL

CATEGORIA III - PERCURSO SOCIODEMOGRÁFICO DURANTE A CARREIRA

1) **Enquanto jogou futebol acumulou alguma outra atividade profissional? Se sim, qual e como conciliou as duas atividades?**
2) **Quantos anos viveu, enquanto profissional de futebol, fora da sua área de residência habitual?** (considerando a sua morada oficial)
3) **Qual a evolução do seu agregado familiar ao longo da carreira?** (momento em que se casou/juntou; filhos; divórcio; etc...)
4) **O agregado familiar acompanhou-o (fisicamente) sempre ao longo da carreira ou só em alguns momentos? Se não, porque viveu afastado do seu agregado familiar?**
5) **Realizou algum tipo de formação académica, técnica ou afins, enquanto jogava? Se sim, qual(ais)? Se não, porquê?**
6) **A nível socioeconómico, considera que os rendimentos que auferiu ao longo da carreira foram muito altos, altos, médios ou baixos em comparação com o salário médio do país?**

CATEGORIA IV - PERCURSO EPIDEMIOLÓGICO DURANTE A CARREIRA

7) **Quais as principais lesões que teve durante a carreira. Qual(ais) foi(ram) reincidente(s)?** (lesões que continuaram a persistir durante a carreira)
8) **Realizou alguma intervenção cirúrgica ao longo da carreira? Se sim, qual(ais) e quando?**
9) **Teve um bom acompanhamento médico-desportivo ao longo da carreira? Porquê?**
10) **As metodologias de treino que vivenciou tiveram preocupação com a sua saúde física, mental e/ou social? Se sim, em que aspetos? Se não, porquê?**
11) **Teve apoio psicológico durante a carreira? Se sim, em que moldes e qual a importância? Se não, porquê?**

ÁREA 3: A TRANSIÇÃO PARA O PÓS-CARREIRA

CATEGORIA V – O MOMENTO DO ABANDONO DA CARREIRA

12) **Qual foi a principal causa para o término da sua carreira profissional?** (idade; lesão; falta de proposta profissional; outra)
13) **Foi fácil ou difícil a decisão de abandonar a carreira profissional? Porquê?**
14) **Planeou o momento do abandono da carreira profissional? Se sim, quanto tempo antes? Alguém o incentivou/influenciou a tomar essa decisão? Se não, porquê?**
15) **Quais as pessoas mais importantes no momento da transição para o pós-carreira? Em que aspetos é que essas pessoas o ajudaram?**

CATEGORIA VI - PERCURSO SOCIODEMOGRÁFICO NO PÓS CARREIRA

16) **Quais foram as experiências/adaptações mais difíceis após o final da carreira profissional?** (a nível familiar, profissional, económico, outras ...).
17) **Após o término da carreira exerceu outra(s) profissão(ões) / atividade(es)? Se sim, qual(ais)? E quanto tempo após o término da carreira? Se não, porquê?**
18) **Desde o final da sua carreira o seu nível socioeconómico melhorou, manteve-se ou diminuiu, em comparação com o período de jogador profissional? Qual(ais) a(s) principal(ais) razão(ões) para tal?**
19) **O seu agregado familiar alterou-se após o término da carreira e por esse motivo?** (do final até ao momento atual)? Se sim, como é atualmente constituído?
20) **Mantem ligação com o futebol? Se sim, qual? Se não, porquê?** (nenhuma; acompanha apenas como espetador; tem uma função técnica específica não remunerada; tem uma função técnica profissional; dirigente desportivo; outro)

CATEGORIA VII - PERCURSO EPIDEMIOLÓGICO NO PÓS CARREIRA

21) **Sofre ou sofreu de algum(ns) problema(s) de saúde após o término da carreira? Se sim, qual(ais)? E esse(s) problema(s) resultou(aram) diretamente da sua carreira profissional?**

Se sim, como avalia o acompanhamento (médico, psicológico, outros) que tem tido? (muito bom, bom, razoável, mau ou muito mau). **Justifique.**

22) **A sua carreira profissional teve algum impacto direto na sua condição física, mental ou social? Se sim, quais e de que forma isso o afetou ou afeta atualmente.**
23) **Foi submetido a algum tipo de intervenção médica no pós-carreira? Se sim, qual(ais) e o(s) respetivo(s) resultado(s).**

CATEGORIA VIII - PERCEÇÃO SOBRE O PLANEAMENTO DO PÓS-CARREIRA

24) **Considera importante a existência de programas de apoio à transição de carreira (durante e após)? Se não, porquê? Se sim, em que moldes e com que tipos de apoio? Quais são as instituições/personalidades que devem implementar tais apoios/medias? Justifique.**
25) **Tem conhecimento da existência de algum programa de apoio à transição de carreira? Se sim, qual(ais)?**
26) **Que conselhos daria aos atuais jogadores profissionais para prepararem o seu pós-carreira?**
27) **Olhando retrospetivamente para a sua carreira, momento de transição e pós-carreira faria algo de diferente? Se sim, o quê?** (exemplos a dar em qualquer um dos 3 momentos: decisões profissionais; decisões pessoais/familiares; abusos/dependências de álcool, drogas ou outras substâncias; estilo de vida; outros).
